# A low-cost alternative method of generating fibronectin micropatterned lines for cellular applications^✰^^✰✰^

**DOI:** 10.1016/j.mex.2023.102240

**Published:** 2023-06-01

**Authors:** Johanna Elisabeth Becher, Franziska Lautenschläger, Divyendu Goud Thalla

**Affiliations:** a Centre for Bioinformatics, Saarland University, 66123 Saarbrücken, Germany; b Experimental Physics, Saarland University, 66123 Saarbrücken, Germany; c Centre for Biophysics, Saarland University, 66123 Saarbrücken, Germany

**Keywords:** *A low-cost alternative method of generating fibronectin micropatterned lines for cellular applications*, Micropatterning, Patterned lines, Fibronectin coated lines, 1D migration

## Abstract

The cellular microenvironment contributes to the architecture, differentiation, polarity, mechanics and functions of the cell [1]. Spatial confinement of cells using micropatterning techniques allows to alter and regulate the cellular microenvironment for a better understanding of cellular mechanisms [2]. However, commercially available micropatterned consumables such as coverslips, dishes, plates etc. are expensive.

These methods are complex and based on deep UV patterning [3,4]. In this study, we establish a low-cost method for effective micropatterning using Polydimethylsiloxane (PDMS) chips.

• We demonstrate this method by generating fibronectin-coated micropatterned lines (width, 5 µm) on a glass bottom dish.
• As a proof of concept, we culture macrophages on these lines. We additionally show that this method allows to determine the cellular polarity by measuring the position of the nucleus within a cell on a micropatterned line.

## Graphical abstract

**Figure:**
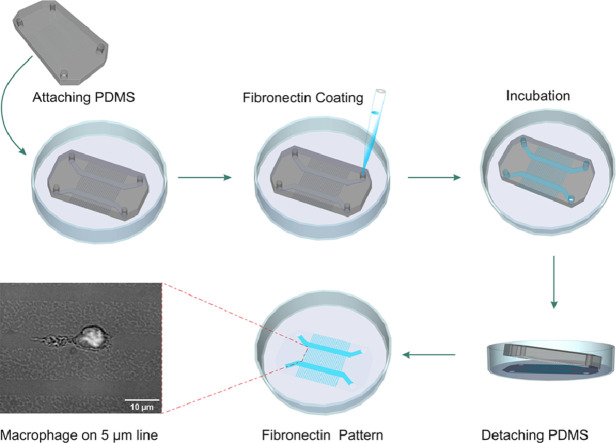

Specifications Table

**Table:** 

Subject area	Physics and Astronomy
More specific subject area	*Biophysics*
Name of your method	*A low-cost alternative method of generating fibronectin micropatterned lines for cellular applications*
Name and reference of original method	*Faure-André, G.,* et al.*, Regulation of Dendritic Cell Migration by CD74, the MHC Class II-Associated Invariant Chain. Science, 2008. 322(5908): p. 1705–1710.*
Resource availability	*NA*

## Method details

### Polydimethylsiloxane (PDMS) chip

**Fig. 1:**
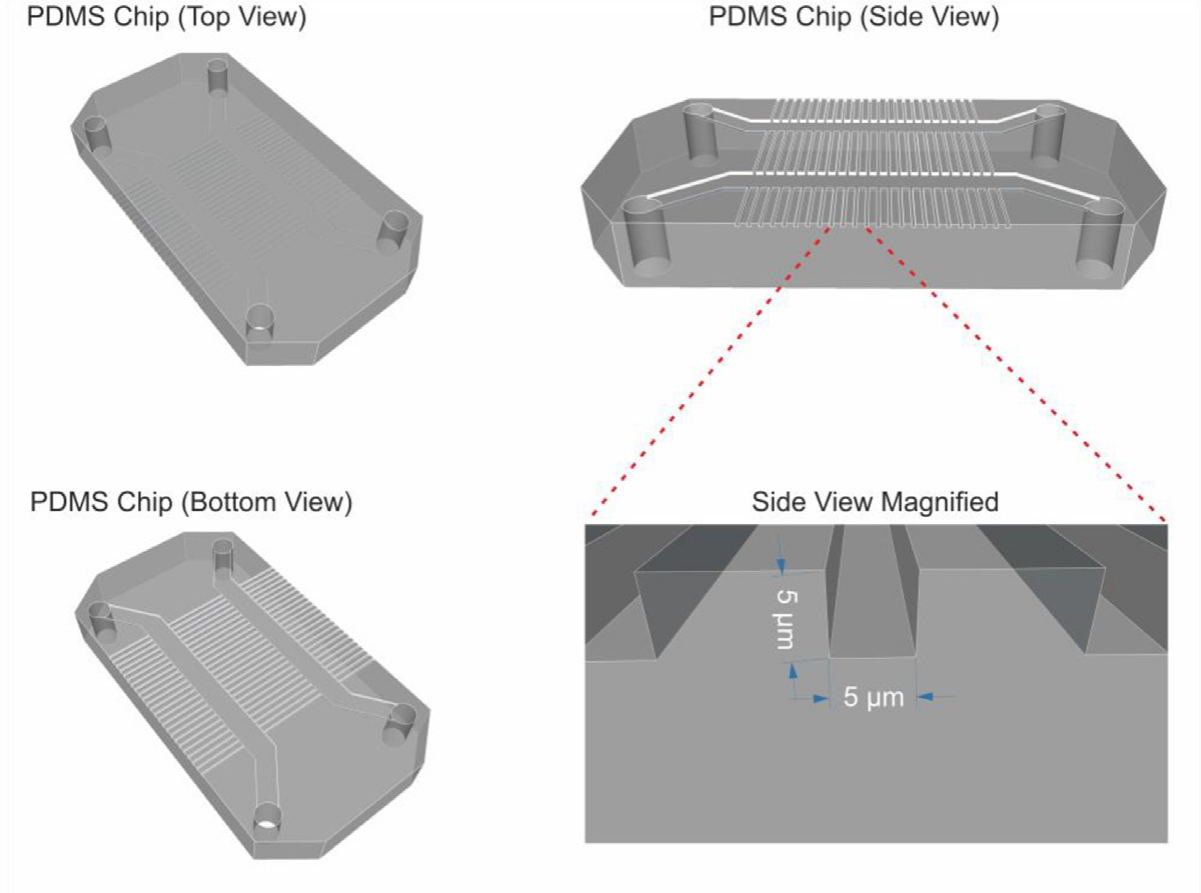
Illustration of PDMS chip with 5 µm channels.

Preparation uses an existing PDMS microchip with 5 µm wide channels (Fig. 1) [5,6].

### PDMS chip attachment

• Place the glass bottom dish (WPI's FluoroDish, 35 mm Diameter, 23 mm Well, Petri Dishes) and PDMS chip facing upwards into a plasma cleaner (diener, Germany) and activate the surfaces for 1 min.

Note: If you don't have access to plasma cleaner, you can make a soft PDMS chip by mixing the PDMS (RTV 615 kit, Momentive performance materials, US) and curing agent in a ratio of 30:1. This chip is sticky and it can be attached directly to the glass bottom dish / coverslip.

**Fig. 2:**
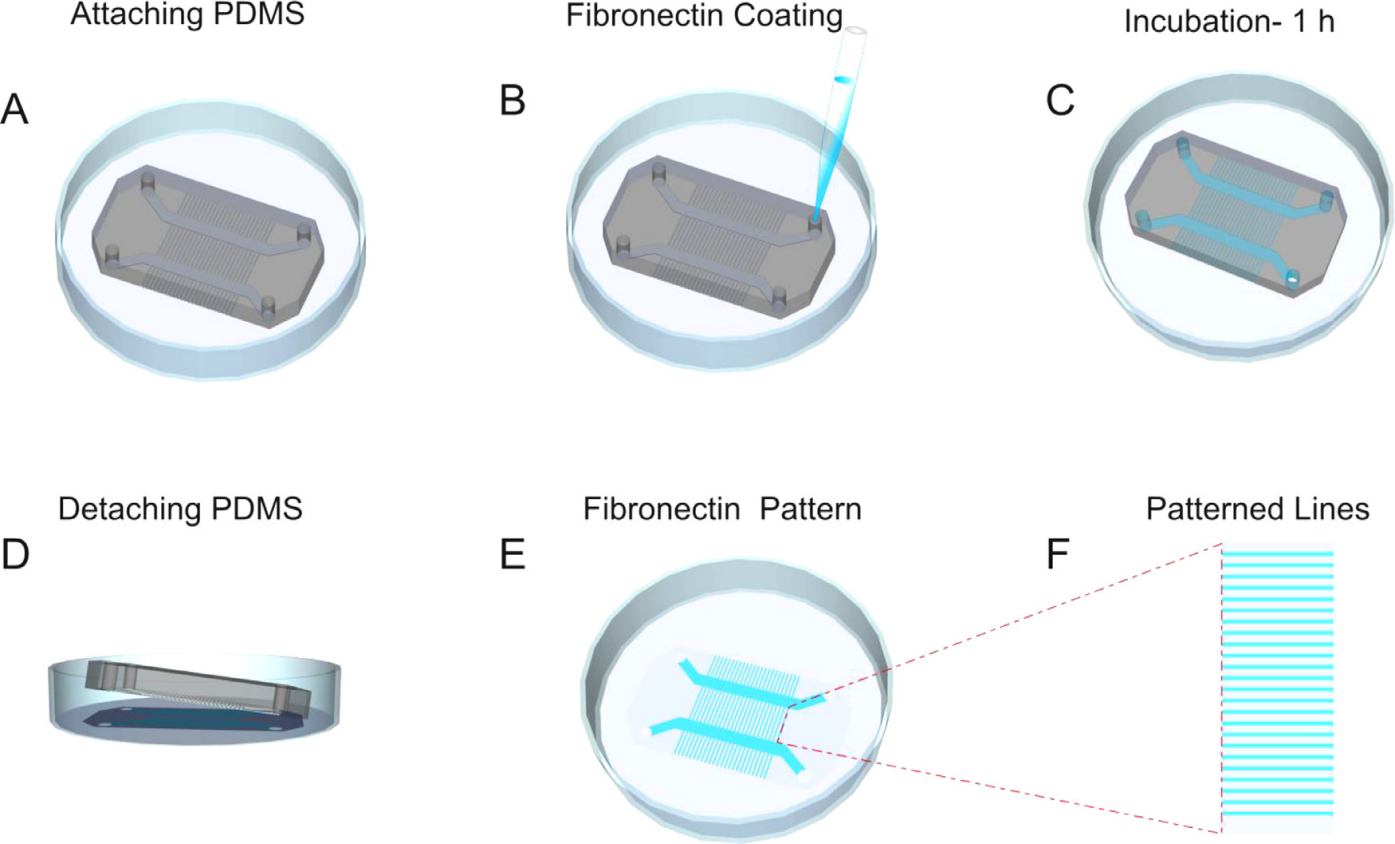
Stepwise demonstration of generating fibronectin-coated lines on glass bottom dish.

• Remove the chip and dish from the plasma machine and attach them by applying a gentle press.
• Now the PDMS chip is attached to the glass bottom dish (Fig. 2A).

Fibronectin Coating:

• Load 10 µl of fibronectin solution (25 µg/ml, Sigma) into each hole of the PDMS chip (Fig. 2B).
• Seal the dish with parafilm to avoid drying of fibronectin and incubate for 1 h at room temperature.
• Peel off the PDMS from the glass bottom dish (Fig. 2C).
• Wash the dish 2 times with phosphate-buffered saline (PBS).
• Fibronectin-coated 5 µm lines are generated on the glass bottom dish (Fig. 2E, F).

## Method validation

To validate our method, we added 500 µl of macrophages (4000 cells/µl) onto the glass bottom dish containing 5 µm wide fibronectin-coated lines and incubated them for 1 h at 37 °C. Next, the dish was washed once with PBS to remove the unattached cells and fixed with 4% paraformaldehyde (Science Service) for 10 min. Then, the sample was mounted using Fluoromount-G™ Mounting Medium with DAPI (Invitrogen). The images of macrophages with nuclear stain DAPI on the fibronectin-coated lines were acquired using a fluorescence microscope (Ti-Eclipse, Nikon).

**Fig. 3:**
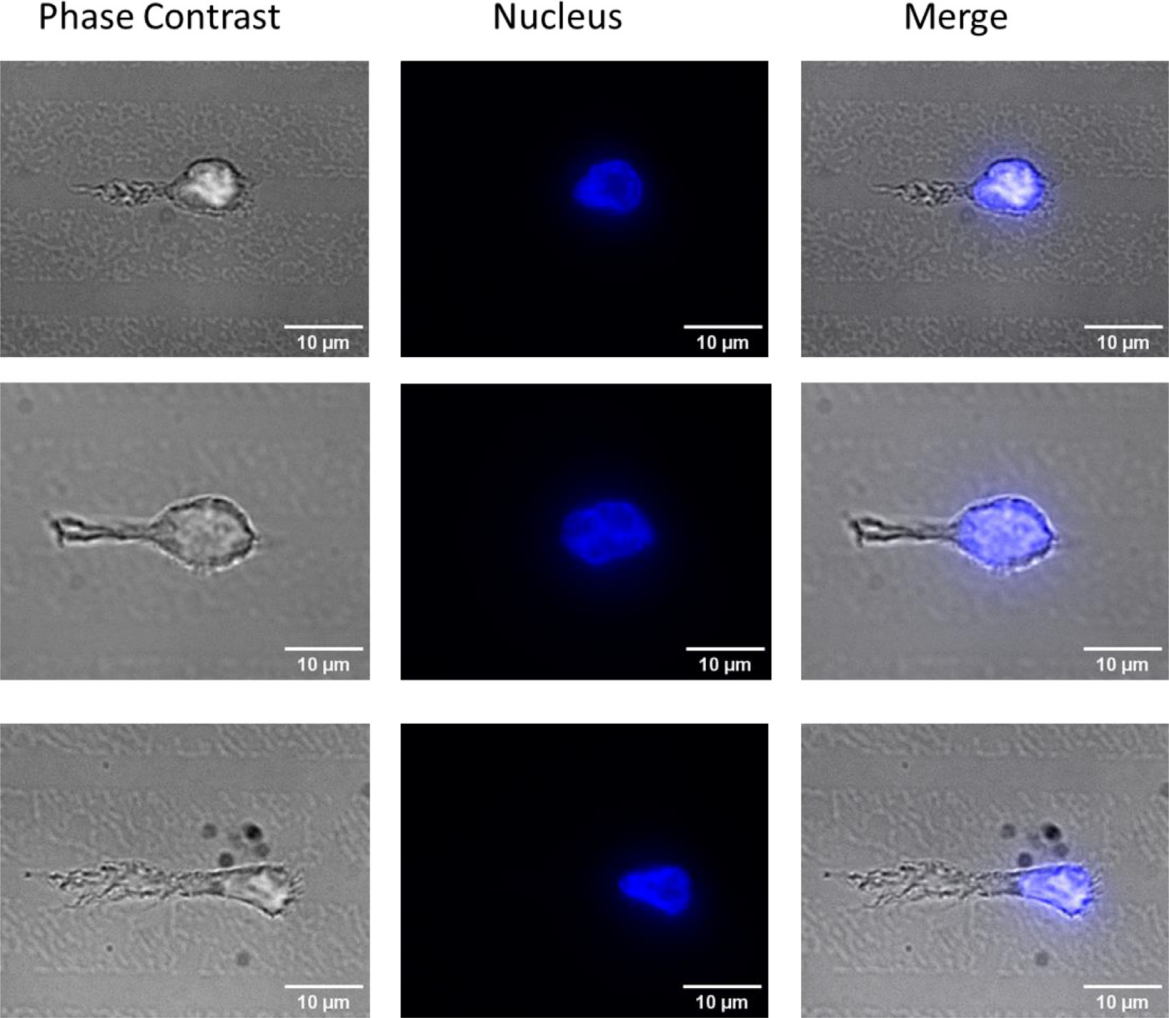
Macrophages adhered to fibronectin-coated lines. The nucleus is stained with DAPI (Blue).

From the data, it is evident that the macrophages adhere to the fibronectin-coated lines (Fig. 3) and a polarized localisation of the nuclei can be observed. We implemented this method in a recent publication where we show the secretion of extracellular vimentin at the back of activated macrophages [7].

Altogether, we successfully demonstrated a low - cost method to generate fibronectin-coated lines which can be easily prepared in any laboratory. This method has a potential to be used in 1D migration, cell confinement and polarization studies.

In earlier studies, PDMS stamps have been utilized for micropatterning of cells, but they focused on generating patterns on the PDMS material rather than directly on the glass substrate [8]. Alternatively, in other studies, PDMS peeling micropatterning techniques have successfully produced micropatterns; however, these methods often require the use of protein-resistant chemicals for inactivation of the surrounding substrate areas [9,10]. However, in our technique we show the elongation of microphages on the pattern lines without the need of protein-resistant chemicals.

## CRediT authorship contribution statement

**Johanna Elisabeth Becher:** Investigation, Methodology, Visualization. **Franziska Lautenschläger:** Conceptualization, Supervision, Project administration, Funding acquisition, Writing – review & editing. **Divyendu Goud Thalla:** Conceptualization, Methodology, Supervision, Writing – original draft.

## Declaration of Competing Interest

The authors declare that they have no known competing financial interests or personal relationships that could have appeared to influence the work reported in this paper.

## Data Availability

Data will be made available on request.
